# On Gravel, Calculus, and Gout: Chiefly an Application of Professor Liebig's Physiology to the Prevention and Cure of These Diseases

**Published:** 1843-04-01

**Authors:** 


### On Gravel, Calculus, and Gout: chiefly an Application of Professor Liebig's Physiology to the Prevention and Cure of these Diseases. By *H. Bence Jones*, M.A., Cantab., Licentiate of the College of Physicians. London: Taylor and Walton. 1842.

The object of this work, as may be seen from the title, is to apply the ingenious principles of Professor Liebig to the palliation and cure of those very distressing affections, gravel, gout, and calculus. It certainly will be admitted that the chemists have, within the last few years, made splendid contributions to the elucidation of some of the most formidable diseases to which flesh is heir; and we are free to acknowledge that we have not the slightest objection to their considering and treating the human frame as a chemical laboratory, provided always it be kept in view that it is a *living* one. We shall without further preamble introduce our author. He commences by describing the prominent character of the *uric acid diathesis*.

### CHAP. I.—ON THE URIC ACID DIATHESIS.

Of the substances produced within the body, and which, as being of no use, are to be eliminated, it may be laid down, that acids, highly nitrogenised substances, and salts, are carried away by the kidneys. In the healthy state the excess of water in the body thrown off with them is sufficient to hold them in solution, and it is only when the nitrogenised substances are produced in excess, compared with the water, or when they do not undergo the ordinary changes, or when the acids which effect the solution are wanting, that a deposit takes place in the urine. The deposit may be either in the form of a white powder, that is, without definite form, or with a definite crystalline form. The substances without definite form, and rendering the water thick and muddy, consist of urate of ammonia and phosphate of lime, with phosphate of ammonia and magnesia. Those with a definite, crystalline mass, or gravel, alone or mixed with the powdery deposit, consist of uric acid, oxalate of lime, and phosphate of ammonia and magnesia. The deposit assumes different colours, according to the substances with which it is mixed; the substances which form urinary concretions, when pure, being perfectly white. By uric acid diathesis is understood that state of the system which produces a constant deposit in the urine of uric acid, either free, or combined with some base. This is generally red, sometimes yellow, most rarely white, entirely soluble in alkalies, and by heat alone, when it consists of urate of ammonia. It may take place in health occasionally—its constant occurrence, however, indicates disease.

In the Second Chapter our author considers *the Changes which take place in the Albuminous Tissues*. " We can," he says, " have no idea of life continuing without perpetual change, which is evident from the never-ceasing muscular action, respiration, secretion, and absorption. The constant product of uric acid is a consequence of these changes. The albuminous tissues gradually divide into different substances, some useful, others useless, and these last are removed from the body, though for their more easy removal they may, as in the case of uric acid, undergo further changes. At present we must consider the uric acid as arising directly from the albuminous tissues, that is, without any other substance being previously formed, from which the uric acid is afterwards produced. We have as yet no proof of a previous stage." In order to account, in some degree, for the causes which hasten or retard the changes which occur in these albuminous principles in the body, it becomes necessary to adduce a statement of Professor Liebig's views on this part of the subject.

The cause of change in the albuminous substances is the chemical action of oxygen, which takes place only when the resistance which the vital force of living parts opposes to this chemical action is weaker than that chemical action itself.

This vital force requires for its manifestation a high temperature, and is diminished by muscular exertion, so that the amount of change in the albuminous tissues may be taken to be directly proportional to the amount of muscular action, the times and temperatures being equal; and in equal times, with equal muscular action, if we surround a part of the body with ice or snow, there occurs more or less quickly, in consequence of the loss of heat, an accelerated change of matter. Such change may be promoted indirectly by accelerating the formation of the ultimate products from these tissues—by increasing the action of the oxygen, by increasing the blood-globules, and by abstaining from non-azotised food, by giving an excess of albuminous food, and by giving an alkali to promote the formation of bile. The oxygen of the atmosphere is the proper active external cause of the waste of matter in the animal body ; it is a force which tends to destroy the vital force at every moment. The resistance which the vital force makes to its action is very different at different stages of life, and varies with the different states and circumstances in which the body is placed. From all this it appears, that the less resistance the vital power makes to the action of oxygen, the greater will be the amount of change, and the more uric acid will be produced in the albuminous tissues; the resistance being diminished by muscular exertion and low temperature, an excess of uric acid will also be produced on that account. Increasing the amount of oxygen and thereby overcoming the resistance will be productive of the same effect. Thus, much oxygen, a low temperature and great muscular exertion will occasion an excessive production of uric acid from the albuminous tissues. From this it appears the indication should be to increase the resistance of the vital force and to lessen the action of oxygen ; this is to be accomplished by avoiding everything tending to diminish the vital principle and by tonics ; another and a more important indication is, however, also to be attended to, viz. to promote further change in those substances which arise from the tissues, that is, to effect a change in the uric acid itself; a deposit containing uric acid can rarely occur as a consequence of excessive changes in the albuminous tissues, for in such cases a large quantity of oxygen is generally absorbed, which will effect a further change on the uric acid, that is, uric acid will no longer appear in the urine, but urea, that is uric acid oxidized, will be formed in large quantities. The case is different in those carnivorous animals which take in little oxygen, or in which the oxygen cannot act on the uric acid in consequence of an excess of non-nitrogenous food being eaten. We have an instance of this in the boa-constrictor, which makes but little muscular exertion and is kept in a warm atmosphere; here a considerable quantity of uric acid is produced by the changes in the tissues, all of which acid however appears as urate of ammonia in the urine, the small quantity of oxygen absorbed not being sufficient to oxidize it.

With respect to the changes, which occur in the gelatinous tissues, our author comes to the conclusion that, at least, whenever an insufficient quantity of oxygen is present, uric acid may be produced by the metamorphosis of the gelatinous tissues. He now considers *the change which the uric acid undergoes after its formation.* Some of the changes which uric acid may be made to undergo out of the body are thus stated by Liebig: when uric acid is subjected to the action of oxygen, it is first resolved, as is well known, into alloxan and urea; a new supply of oxygen acting on the alloxan, causes it to resolve itself either into oxalic acid and urea, or into oxaluric and parabanic acids, or into carbonic acid and urea; that is, if a full quantity of oxygen is given to the uric acid, carbonic acid and urea may be obtained; if a smaller quantity, oxalic acid and urea; and if none is given the acid remains unchanged.

Such changes may be effected in the laboratory, by submitting uric acid to heat, oxygen and water; and it is by these three agents that similar changes are effected in the body. Long exposure of impure urate of ammonia to the action of the oxygen of the atmosphere seems to change the uric into oxalic acid. The oxygen is by far the most important agent in these changes.

Liebig has shown that the waste of matter and the production of animal heat are both dependent on the absorption of oxygen. This is necessary to furnish its elements for the production of alloxan and urea, and also to keep those substances which are formed in such a state as may enable the oxygen to act on them most readily, whilst the heat renders the chemical changes more rapid and more complete. In health these agents are sufficient to change all, or nearly all, the uric acid; but at other times they may not be in sufficient quantity, especially the oxygen; so that, when there is want of change in the uric acid, it indicates in almost every case a deficiency of oxygen, and this may in general be regarded as the cause of the uric acid diathesis.

We may now see on what the quantity of uric acid actually thrown out of the body depends; as it is not possible for the uric acid to vary, excepting, 1st, with the quantity produced in the body; 2nd, with the quantity changed before it is thrown out; and supposing none to be changed, or the quantity changed to be always the same, then the quantity thrown out by the urine will vary directly as the quantity produced by the metamorphosis of the tissues. If, on the contrary, we suppose the quantity produced to be constantly the same, then the quantity thrown out will vary inversely as the quantity which undergoes a change in the body; because the more uric acid is changed, the less will remain to be thrown out.

The author's endeavour has been to shew that, in case of an excessive production of uric acid in consequence of a rapid change in the tissues from an excessive quantity of oxygen, as the excess of uric acid will be acted on and changed by the excess of oxygen into urea and carbonic acid, no deposit will appear in the urine under such circumstances; hence we have to consider only the quantity which is changed in the body, as influencing the quantity of uric acid in the urine; this general law our author thus states: the quantity of uric acid thrown out of the body varies inversely with the quantity which undergoes a change in the body. Of all the agents which effect this change we have seen that oxygen is the most necessary; whence it may be concluded that the quantity of uric acid thrown out varies inversely with the amount of the action of oxygen, and thus, with the exception of tonics, the problem of curing the uric acid diathesis depends on this, viz. how the uric acid in the body can be most acted on by oxygen, how it can be changed into urea and carbonic acid: our author says that this may be done—

1st. By giving a large supply of oxygen, as by exercise, by cold air, and by medicine, as by nitrous oxide water, and iron.

2dly. By diminishing the quantity of other substances, on which the oxygen acts more readily than on the uric acid; that is, substances consisting of hydrogen, carbon, and oxygen only:—as by abstaining from these as food, by removing them by aperients, and by sudorifics.

3rdly. By keeping all the uric acid produced in solution, by water and by alkalies. 1st. An increased amount of oxygen may be taken in by exercise, the consumption of oxygen in equal times being represented by the number of respirations. The same end may be attained by cold air, though in a less degree. The use of iron is also recommended for the same purpose, this mineral having the power, according to some, of increasing the number of red globules. Hence iron may be supposed to be an agent in increasing the amount of oxygen which is taken to the capillaries. 2ndly. The action of oxygen on the uric acid may be increased by lessening the compounds of hydrogen, carbon, and oxygen in the body; and this may be done by abstaining as much as possible from that food called by Liebig non-nitrogenised, or the "elements of respiration." These substances, serving for the support of animal heat, readily combine with oxygen, and thus, when they exist in large quantities in the body, they hinder the action of the oxygen on the uric acid, which would otherwise be changed into urea and carbonic acid. So that the existence of these substances in the body in large quantity prevents the uric acid from being oxidized. Starch is well known to constitute a considerable part of our food. When taken into the stomach it begins to change into sugar, and by degrees the whole is rendered soluble. This continues usually a few hours after a full meal, and therefore, during this time, the quantity in the blood is larger than at other times. Hence probably it is, says our author, that there is less action on the uric acid, and we consequently find the greatest deposit of uric acid, or the urates, takes place after a large meal and continues for some hours. The oxygen may act on the sugar and fat, and convert them into carbonic acid and water, or these substances may first undergo some further change before they combine with the oxygen.

The next means which we possess for increasing the action of oxygen, is by diminishing the quantity of those substances on which the oxygen acts, by aperients, and particularly by those medicines which promote a secretion and evacuation of bile. Whatever the theory be, which we may adopt, the fact is certain that by purgatives we lessen the quantity of carbon in the blood, and so permit a freer action of oxygen on the uric acid. Emetics which produce bilious vomiting are capable of producing the same effects. The quantity of non-nitrogenous compounds may also be diminished by sudorifics. The perspiration contains both acetates and lactates, which are compounds of carbon, hydrogen, and oxygen. By sudorifics these substances are removed from the blood, and thus the oxygen, which would have been necessary to convert these into carbonates, is left to act on the uric acid.

3rdly. The uric acid may be acted on in an increased degree, by keeping it in solution; and this must be done from the time of its production in the ultimate tissues; for this purpose free alkalies, alkaline carbonates, salts of the vegetable acids have been found useful.

We now come to the *treatment of the uric acid diathesis*. First, with respect to *exercise*—this should always be regulated by the amount of fatigue produced. Exercise producing perspiration is the most beneficial, and this the more so the colder the air is; as a greater quantity of oxygen is absorbed thereby; we must beware, however, of depressing the vital powers, by which an excess of uric acid might be produced. Sleep should be indulged in only so far as may be necessary to repair the fatigue which exercise has produced. Hot rooms should be avoided. The use of nitrous oxide water has been recommended as the best diluent. By the various preparations of iron we may encrease the amount of red particles in the blood and thus influence the quantity of oxygen which is absorbed—the sesqui-oxide of iron has been deemed the best; and as, according to Liebig, it is in this state the iron exists in the red-colouring matter of the blood, there may be some grounds for considering this the best preparation of iron. To facilitate its absorption it should be given in the minutest state of division. For this purpose it should be given newly precipitated from some soluble salt of iron, as from the sesquichloride or persulphate of iron, from which the hydrated peroxide of iron may be formed by adding carbonate of ammonia or soda.

Our author now considers the treatment by diminishing the non-nitrogenous principles in the blood. This treatment he founds on the principles contained in Liebig's Physiology:—It has been shewn, he says, that the substances which contain no nitrogen, by combining with the oxygen which has been inspired, hinder the action on the uric acid; and it is highly probable that no albumen undergoes metamorphosis until it has served the purposes of life. These are the first principles by which the practice should be governed; and hence by far the most beneficial diet is a moderate quantity of meat, with a much smaller quantity of bread. The kind and quantities of both must be regulated by experiments and by consideration of the habit and exercise of the patient. The quantity of starch in flour, as compared with animal food, renders it unsuitable to live only on bread. Meat alone would be far more beneficial; but in most people the process of respiration almost requires some substance which contains no nitrogen, though it should be taken in small quantities only. If the quantity of bread be small, the quantity of animal food may be proportioned to the exercise and the state of the body; for thus the changes which are going on in the tissues will be repaired, and the oxygen, finding but little non-nitrogenous substance in the blood, will act more readily on the uric acid, changing it into urea and carbonic acid. Sugar and starch comprehend much the largest part of those substances in vegetables which can be absorbed; nitrogenous and oleaginous substances are present generally in small quantities, though the relative amount of these principles varies much in different species. Thus, potatoes and rice are amongst those in which most starch is found, and these are therefore most inadmissible, while in greens and peas, there is much more nitrogenous matter, which in peas is similar to cheese. Fruits usually contain large quantities of starch and sugar; on this account apples and pears are most objectionable.

Among non-nitrogenous substances we must include fat. If the formula for this is taken, as $C_{11}\ H_{10}\ O$, then 31 equivalents of oxygen are required to convert this into carbonic acid and water, and by taking this substance as food, so much oxygen is prevented from acting on the uric acid. Neither should butter be taken in excess. Gelatine, which forms the principal constituent of soups and jellies, may be used as a partial substitute for meat; but as the albuminous tissues cannot be formed from it, it cannot be entirely substituted for it without the strength failing.

With respect to drink, the oxygenated water is best, then water which has been distilled, as being the best solvent. This, however, cannot be had everywhere, Some recommend the water to be filtered. This process removes all the substances suspended, but not those dissolved in it. Boiling the water has also been recommended.

We have already seen that the non-nitrogenous bodies in the blood may be diminished by aperients which act on the liver. These are most useful when the deposit is dark-coloured. Of such medicines, calomel, aloes, colchicum, and colocynth are beneficial, both in large and purgative doses, and also when given in such a way as to increase the secretion of the liver. Hence the efficacy of blue-pill as an alterative.

Sudorifics are sometimes given with advantage. With respect to baths, their action may be considered on the nerves and on the blood, and on each the action is of two kinds; thus, on the nerves there may be a stimulant or a sedative action, and on the blood they are capable of removing substances from it and of enabling them to be absorbed into it. These modes of action depend on the state of the system, the temperature and the substances which are dissolved in the bath. In one point of view, the skin may be looked on as an expansion of the nerves of sensation, and by acting on the nerves of sensation, the whole system may be influenced. The action of the bath on the nerves of sensation depends on its temperature, as well as on the state of the system; thus, in some states, a warm bath causes irritation, and in others, it acts as a sedative, whilst the same is the case with cold applications.

The removal of substances from the blood depends also on the temperature of the bath and the state of the system, and for this the vapour bath is most efficacious. The absorption of substances by the skin, does not seem to depend on the temperature, but on the state of the system, and on the substances which are contained in the bath. A great part of the benefit derived at Teplitz by those subject to the gout, depends on the absorption of alkalies by the skin.

As it cannot be expected that there will be the same amount of absorption when the system is saturated with moisture, as when a large quantity has been removed by the secretions during the night, it has been directed to take the baths early before food. Our author here suggests two kinds of baths to be used in immediate succession. This is the vapour bath, to remove water and acid substances from the blood, and immediately after an alkaline water bath, by which alkaline water may be taken into the blood. The next point to be attended to, is to keep all the uric acid in the ultimate textures in solution. This may probably be effected by water and alkalies. When these, or their carbonates, are given, they should be taken and dissolved in water at least an hour before food, so as not to interfere with digestion; these medicines may relieve the complaint, but they can never cure it.

When uric acid is deposited in crystals, which may be recognised by their insolubility by heat, when they are soluble in an alkali, alkalies are then required, and this medicine must be given in such quantities as to combine with all the uric acid formed, when the deposit will either disappear altogether, or be changed for the urate of ammonia. By carefully abstaining from all vegetable acids, a crystalline deposit of uric acid will scarcely occur.

Our author makes a remark here, on the effect of salt in promoting the action of oxygen on the substances in the body, viz. that no animal can be fattened, if it is able to obtain much salt. When sailors are long fed on salt meat, which furnishes an abundant supply of alkali to the blood, whilst they are deprived of non-nitrogenous food, an excessive action of oxygen on the tissues of the body takes place, and when this is long continued, the scurvy appears, the salt hastening the rapid changes in the tissues. By diminishing the amount of alkali in the blood, and by giving non-nitrogenous food, the scurvy is cured or prevented, in consequence of such substances being acted on instead of the tissues of the body. This explains the benefit which arises from vegetable acids, from fresh vegetables, from sugar, wine, beer, wort, treacle, potatoes, &c. all which have been used with the best effects.

We now come to the interesting subject of *Gout*, which our author holds to be the invariable result of the long continuance of the uric acid diathesis; according to Liebig's views, it is most probable, he says, that great weakness, and inordinate muscular exertions, may increase the amount of metamorphosis in the tissues, and thus give rise to new substances, which must be oxidised in order to be removed, whilst excessive eating, sleeping, and want of exercise, constipation, vegetables, and little secretion by the skin, all produce the same effect,—all lessen the amount of oxidation which is necessary to remove those substances which result from the metamorphosis of the tissues of the body. Physicians of all ages have insisted on several of these as the cause of gout, and have recommended the opposite of them, as temperance, early rising, moderate exercise, alkalies, and medicines which act on the liver and the skin, as the means of cure. In the language of Professor Liebig, they have declared that the want of oxygen is the cause of gout, and the promotion of its action the only means of effecting a cure, or of hindering a return of the complaint. Numerous authorities are adduced by our author in support of these causes of gout. In Austria, a mode of treatment has been revived, which, in those who can endure it, is most beneficial in the diseases which may be included in the uric acid diathesis, as indigestion, bilious complaints, gout, rheumatism, and skin diseases. At Gräffenburg, in Austrian Silesia, under Priesnitz, the action of oxygen is promoted to a most beneficial extent in these diseases, but to a no less disastrous one in the opposite class of diseases which arise from too much action of oxygen on the body, as in phthisis and scorbutic cachexia. Until Professor Liebig again directed attention to the action of oxygen on the human body, the causes of success and failure were unknown. At Gräffenburg the greatest possible action of the skin is produced by baths. Large quantities of water are required to be taken. Early rising, and a plain, though not strict diet is ordered. By these means, the action of oxygen on the body is promoted to a very great degree; "and death ensues if ever the system is no longer able to furnish matter to resist the action of oxygen;" the condition of health consisting in an equilibrium among all the causes of waste and supply; "and death being that condition in which all resistance on the part of the vital force to the action of oxygen entirely ceases."—*Liebig.*

Much of the treatment at Leamington, and the benefit derived from it, may be accounted for in the same way. At first, abstinence and drastic medicines are ordered, and the strictest diet is enjoined, which consists chiefly of meat, with a small quantity of bread, and no fermented liquids, and much exercise. One hour's walk, at the least, before each meal. Large doses of iron are afterwards given. The waters, containing a considerable quantity of common salt, chloride of calcium, Glauber's salt and traces of iron, would assist the more energetic measures of Dr. Jephson, in promoting the action of oxygen in the body, as is soon indicated by the altered complexion.

Many of the German baths, as Teplitz, Marienbad, Carlsbad, Kissengen, Emms, and Weisbaden, are beneficial in gout, but not so much as the above plans of treatment, because the privations are not so great, and the other means used for promoting the action of oxygen are not so heroic: however, in the composition of the waters themselves they are superior. It appears that the drinking of alkaline or earthy carbonates, with small quantities of iron, as well as warm baths of the same waters, early rising and exercise, temperance, simple diet, and warm clothing, are nearly all the principal means of promoting the action of oxygen on the body. Excepting purgative medicines and active exercise, they include all the practice laid down by our author in the treatment of the uric acid diathesis. At Teplitz nearly two-thirds of the visitors suffer from gout. The waters drunk are the gartenquelle (cold), and the hauptquelle (hot). The first contained, when 10 pounds of water were evaporated to dryness, 47 grains of solid residue, of which 28·9 grains consisted of carbonated alkalies and earths, with a trace of iron. The second spring contained 48·4 grains of solid residue, of which 32·1 are carbonates. Both springs also contain chlorides of sodium and potassium, and small quantities of sulphates of potash and soda. From three to six goblets of these alkaline waters are drunk, with intervals of a quarter of an hour between each glass, commencing between six and eight in the morning. The last of these springs and others of nearly the same constitution, are used as baths; as complete baths, as well as hip or foot-baths, during from fifteen minutes to an hour before breakfast. By this means the amount of absorption is increased. After the bath, perspiration is promoted—temperance and simple diet are also prescribed.

At Teplitz, the alkalies are absorbed by long-continued baths, rather than by much drinking, and but little purgative salts are contained in the waters, whilst the country around is not so attractive to those who take walking exercise as to those who ride or drive. Hence those in whom the gout is connected with obstructions of the liver will gain more advantage from more purgative mineral waters, and those who are able to take exercise on foot will find more inducements to it in other watering-places; where also more alkali is absorbed in consequence of more water being drunk, and where the excretory action of the skin is a consequence of exercise. Hence at Teplitz, those who are weak and unable to take exercise by walking, or to endure strong purgatives, will find the greatest relief; while those in whom the gout is accompanied by obstructions of the bowels or liver, and who are strong, will find more benefit at Marienbad, Carlsbad, or Kissengen.

Professor Liebig's views of gout are, that it is an inflammation in parts in which the usual changes, which the oxygen effects, are unable to take place, in consequence of an excess of the non-nitrogenous principles in the body; whilst rheumatism or "the universal gout" seems to arise from the changes being checked by the action of cold on the skin. There are however some points respecting gout and rheumatism which still require to be determined before the whole of the phenomena of these diseases can be perfectly explained. One is the question whether the uric acid is, in the state of health, produced in the ultimate textures, and there undergoes its changes into urea, in which state it is carried to the kidneys by the circulating blood, (and the existence of urea in the blood in Bright's disease and in cholera is in favor of this view,) or whether in the ultimate textures some other substance is first formed from the tissues, which, when it arrives at the kidneys, is there changed into uric acid, and afterwards into urea, in the way that has been pointed out. If this last be the case, the substance out of which the uric acid is formed must exist in the blood; and it is, our author observes, amongst those substances which are comprehended under the term, indefinite extractive substances of the blood, that search must be made for it. The probability of some such substance existing is increased by the most accurate examination having as yet failed to detect uric acid or urea in healthy blood.

"Certainly," says our author, "the one or other of these states must exist; either there is an excess of that substance which is capable of producing uric acid when it comes to the kidneys, which is passed during an attack of gout from the systemic capillaries to those of the kidney, and there gives rise to the excess of uric acid which appears in the urine, or, what is perhaps more probable, urate of ammonia itself (formed from the metamorphosis of the tissues) exists in the blood in larger quantities than usual. One or other must exist in the blood, during the fit of the gout, in larger quantities than in the state of health, and from one or other the excess of uric acid appears in the urine, and most probably it is the presence of this substance in the circulation, and the interruption by any causes, as cold and weakness of circulation, of the changes which it is undergoing in the capillaries and in the kidneys, that produces the phenomena of retrocedent and irregular gout. The acid state of the secretions of the skin, stomach, and urine in this disease, has led some physicians to consider acidity as the cause of this disease; such a state of acidity may also arise from a want of action of oxygen on the non-nitrogenous principles, that is, from the oxygen not being present in sufficient quantities. In health, when sufficient oxygen is absorbed, the acids formed from the non-nitrogenous food are converted into carbonic acid and water by combining with oxygen; but in gout the deficiency of oxygen renders those changes impossible, and the acids formed, being but very imperfectly or not at all acted on by oxygen, remain in the body undecomposed till they are thrown out in every possible way. By soda, potash, or magnesia taken as medicine, these acids are neutralised, as well as by the carbonates of those alkalies which are decomposed, lactates or acetates being formed. Hence the benefit from alkalies in gouty complaints, but it is only palliative. Such treatment, however, will not prevent the formation of lactic acid; to effect which, all the substances from which this acid most readily arises, especially starch and sugar, should be left off.

" By this action of oxygen, then, an explanation is afforded of the effects of weakness, excessive sleeping, excessive eating, of a want of exercise, as well as of inordinate muscular exertion, of vegetable acids, and of alcohol in the production of gout. For the weakness and inordinate exercise tend to the excessive production of uric acid, disposing to and causing rapid changes in the tissues. Sleep lessens the quantity of oxygen which can be absorbed, by lessening the number and the strength of the respirations, and in consequence the uric acid formed is not changed. By excessive eating the blood becomes loaded, and contains an excess of those principles which can only be carried off by the action of the oxygen. By acids the uric acid is rendered less soluble, and therefore less easily acted on. Wines are injurious from the alcohol they contain, and acid wines are doubly injurious by both acid and alcohol adding to the excess of non-nitrogenous substances, for which the oxygen has a greater affinity than it has for the nitrogenous substances."

The juvantia give additional strength to this view. We see abstinence alone will cure by giving no fresh fuel, the supply of oxygen remains undiminished and gradually consumes those non-nitrogenous (carbonaceous) substances which had been previously so heaped up as to threaten to put an end to all action. Exertion increases the respiration, and thus causes more oxygen to be absorbed, and thus more action takes place.

Purgatives remove the bile, which contains an excess of carbonaceous matter, on which the oxygen would otherwise act to the hindrance of its action on the nitrogenous substances; whilst alkalies and water hold the uric acid dissolved, and promote the changes in the tissues. By these views the relation of gout to acidity, to uric acid, and oxalate of lime calculi, is most plain. They all arise from the same cause, the want of action of oxygen. The indications of treatment, as suggested by the theoretical views of Liebig, are thus summed up in the words of Schönlein: scil.

1st. To neutralize any diseased products which may be formed.

2nd. In proportion as they are formed, to cause them to be removed, in order to prevent any accumulation which may cause a paroxysm.

3rd. To render the formation of any fresh diseased products impossible.

### On the Oxalic Acid Diathesis; and on the Production of Oxalic Acid from Uric Acid.

If we understand our author here, and we are not quite certain on the point, he says that though from the examination of urinary calculi in different collections, those which contained oxalate of lime bore a very considerable proportion to those in which uric acid was present; and though the former has even been found in some, more frequently than the latter, yet the number of patients who suffered from the oxalic acid diathesis seemed to bear the greatest disproportion to the number of those in whom the uric acid diathesis existed. The intermixture, and alternation also, of oxalate of lime with urate of ammonia or uric acid, was found to be very frequent in calculi. These facts could not be accounted for. The one seemed to point to some intimate relation between these substances; whilst the other seemed to prove that, whenever oxalate of lime was formed, it almost always remained, and produced a calculus. The probability of the first conclusion has been shewn by Liebig. This diathesis has been generally recognized in consequence of a small calculus of oxalate of lime being passed. Now, however, by means of the microscope, the physician may much sooner obtain evidence of the diathesis, which will be rendered all but certain when a substance is obtained which does not effervesce when mixed with a dilute acid, though it does so after a moderate heat has been applied, and again ceases to do so after exposure to a strong heat, with which the ash becomes alkaline, and when dissolved in water gives a white precipitate with oxalate of ammonia.

*On the Production of Oxalic Acid from Uric Acid.*

When mentioning the changes which uric acid undergoes, it has been stated that oxalic acid may be actually produced from uric acid by being subjected to the imperfect action of oxygen; this will in some way prepare us for the apparently odd occurrence of the frequent intermixture in calculi of urate of ammonia and oxalate of lime. In the oxalic acid diathesis, the oxydising process in the body is carried a step further than it is when the uric acid diathesis exists; but it still is stopped short of the extent to which it is carried in the state of health. From this we may deduce rules for the prevention of this diathesis, the object being to increase the action of oxygen on the uric acid, by which it may be changed into carbonic acid and urea. The rules for accomplishing this have been already given. Whilst the treatment of the uric acid diathesis was sufficiently well known, that for the prevention and cure of oxalic acid diathesis was entirely unknown, in consequence of the usual origin of the oxalic acid itself being unknown. Oxalic acid patients were prohibited the use of sugar from the circumstance of nitric acid producing oxalic acid by its action on sugar; other practitioners again prohibited their patients the use of lime. "We can hardly," says our author, "expect any benefit from these rules, when it is known that the usual source of the oxalic acid is from uric acid, and that it is absolutely impossible to abstain from lime in some form or other, as it exists in almost everything which is taken as food and drink. 2nd. Though it is possible that sugar and perhaps other substances of the non-nitrogenous class, may, by imperfectly combining with oxygen in the body, give rise to oxalic acid (and it will be especially interesting to enquire whether this takes place at any time in the course of diabetes, or in diseases related to this, in which there seems to be a want of oxydation of the non-nitrogenous substances), still the oxygen has evidently a much stronger affinity for the non-nitrogenous than for the nitrogenous substances in the body, and thus the process of oxydation is far more frequently incomplete in the latter than in the former; so that we should expect oxalic acid generally to arise from the insufficient oxydation of the uric acid, and much more rarely from sugar; and the alternation of this substance with uric acid in calculi, and the ease with which it is formed from uric acid, leads to the belief that this is the usual origin of oxalic acid. The free oxalic acid passing off by the kidneys meeting with the phosphate of lime, which is secreted both by them and by the mucous membrane of the urinary passages, decomposes it, and oxalate of lime is the result." The same thing happens, when oxalic acid is taken, as such, in the food; if free, like tartaric acid, it passes off at the kidneys, and combines with the lime, which it afterwards meets. If taken in combination with alkalies, like tartaric acid, it would probably be decomposed. Thus, the causes of this disease are generally similar to the causes of the uric acid diathesis, both being referrible to insufficient oxydation and accordingly both requiring similar treatment; there is one point, however, requiring particular notice, and this is the lime. It is evident that, if no lime were taken into the system, no oxalate of lime could be formed, some lime, however, is necessary for the bones and membranes, and it is taken into the system in all solid and liquid food. Now, though it is impossible to obtain food absolutely free from it, and thus to hinder the formation of fresh oxalate of lime, yet by rendering it as free from it as possible, the rapid increase of a calculus may be prevented. Water is the thing most easily purified, and yet it is probably that which contains most lime. So that in the oxalic acid diathesis distilled water should be invariably used. When that cannot be had, rain water should be used. The curative treatment, however, must be directed to the oxalic acid, not to the lime.

### On the Phosphatic Diathesis.

This may be divided into a true and a false diathesis: the true being that in which, in consequence of a general state of the system, the urine becomes alkaline and the phosphates are deposited; the false being that in which alkalescence ensues from an obstruction to the urine, or from the mucus which is secreted, rapidly inducing a change in the urea—as when there is irritation or inflammation of the mucous membrane of the bladder. The false phosphatic diathesis is far more common than the true. In both, the urine is alkaline, and there is a white deposit readily soluble in any dilute acid, but not soluble by heat or alkalies. In this diathesis it is not uncommon to observe a layer of crystals on the surface of the water, which, on exposure to light, presents a varied play of prismatic colours. These consist also of the phosphates.

Phosphorus, phosphoric acid, lime and magnesia, are carried into the circulation of men and animals, partly in their food, and partly in their drink. In these they exist in organic or in inorganic compounds. In vegetable and animal albumen no difference can be observed, even in regard to the presence and relative amount of sulphur, phosphorus, and phosphate of lime. In vegetables we find organic acids combined with lime and magnesia, and in them, phosphates, carbonates, sulphates or chlorides of lime and magnesia are universally present. Most seeds contain certain quantities of the phosphates. There is an abundance of the phosphate of magnesia in the seeds of different kinds of corn. The concretions in the cœcum of horses consist of the phosphate of magnesia and ammonia, the greater part of which is obtained from the oats and bran consumed as food. When the supply of phosphorus, lime, or magnesia is no greater than is required in the body for the bones, the brain, and the membranes, none appears in the urine, as in horses' urine.

The two sources of phosphoric acid in the blood are 1st, from the food, and, 2ndly, from the oxydation of the phosphorus of the tissues; when from these sources an excess exists in the blood, it is thrown out in the urine; and when this is the case there is an additional quantity thrown out by the secretion which takes place from the mucous membrane; for the mucus of all membranes is found to contain salts of lime, and in most cases phosphate of lime. The deposit on the teeth from mucus and saliva contains 79 per cent. of phosphate of lime. This substance is also to be found in the mucus of the bladder and urinary organs. The phosphates, whether derived from the mucus or from the secretion of the kidneys are insoluble in any alkaline fluid; but very soluble in any diluted acid. The urine being, in its healthy state, always acid, these salts are always held in solution, and they remain dissolved until the urine becomes alkaline; which may occur either by taking alkalies, or by the urea becoming converted into carbonate of ammonia. In producing this latter change of the urea the mucus is considered to act as a sort of ferment. In the healthy state this action of the mucus does not occur until after some hours of exposure to the action of the air; but when the secreting membrane is inflamed, the mucus seems to undergo this change much more rapidly.

"The urine is also found to be alkaline, even when it passes from the kidneys in states of great weakness, and after injury of the spinal cord. In weakness, the sulphuric and phosphoric acids, produced by changes in the albuminous tissues, are formed in exceedingly small quantities. These acids are no longer sufficient to convert all the carbonate of lime into sulphate and phosphate, and consequently the carbonate of lime appears in the urine, or is deposited if a neucleus exists. Hence it is, probably, that we so frequently find small quantities of lime in phosphatic calculi. In very great states of weakness, the changes in the tissues are so slow that the smallest quantities of phosphates are produced, and then, though the urine becomes alkaline, still no sensible deposit is found in it. This may be frequently observed in the last stage of Bright's disease, in which, as appears by Dr. Christison's quantitative analysis, the formation of phosphates is greatly diminished; when it will be found that, though cream of tartar may be given so as to render the urine highly alkaline, still it will remain entirely free from any deposit. In injuries of the spine producing paralysis, the urine occasionally is rendered alkaline, in consequence of retention in the bladder giving time for the mucus to undergo its changes. In whatever way the urine becomes alkaline, the phosphates being insoluble in an alkaline fluid, are precipitated, and either form a concretion in the bladder, or are deposited from the urine as a white sediment, which is easily soluble in very dilute mineral acids, and does not disappear even with a strong heat. It is not improbable that, during excessive action of the brain, the phosphorus of the nervous tissues must occasion a great quantity of phosphoric acid."

We have now given a tolerably full analysis of this book, which has been the first attempt to reduce the valuable principles contained in Liebig's Chemical Physiology to practical purposes. We cannot help saying that we regret the author did not put some of these principles to the test of experiment before this work made its appearance. He would thus have recommended it to the profession, by that which is one of the strongest of all recommendations, we mean that of experience. Trusting he will take every opportunity of so doing, and thus remove that air of crudity from the work which it now has, before a second edition is called for, we take our leave of him for the present.

